# The use of electronic alerts in primary care computer systems to identify the excessive prescription of short-acting beta_2_-agonists for people with asthma: a systematic review

**DOI:** 10.1038/s41533-018-0080-z

**Published:** 2018-04-16

**Authors:** Shauna McKibben, Anna De Simoni, Andy Bush, Mike Thomas, Chris Griffiths

**Affiliations:** 1 Asthma UK Centre for Applied Research, Barts Institute for Population Health Sciences, Barts and The London School of Medicine and Dentistry, Queen Mary University of London, London, UK; 2 Asthma UK Centre for Applied Research, Imperial College and Royal Brompton Hospital, Biomedical Research Unit at the Royal Brompton and Harefield NHS Foundation Trust and Imperial College London, London, UK; 3 Asthma UK Centre for Applied Research, Primary Care and Population Sciences, University of Southampton and NIHR Southampton Biomedical Research Centre, Southampton, UK

## Abstract

Computers are increasingly used to improve prescribing decisions in the management of long-term conditions however the effects on asthma prescribing remain unclear. We aimed to synthesise the evidence for the use of computerised alerts that identify excessive prescribing of short-acting beta_2_-agonists (SABAs) to improve asthma management for people with asthma. MEDLINE, CINAHL, Embase, Cochrane and Scopus databases (1990–2016) were searched for randomised controlled trials using electronic alerts to identify excessive prescribing of SABAs for people with asthma in primary care. Inclusion eligibility, quality appraisal (Cochrane risk of bias tool) and data extraction were performed by two independent reviewers. Findings were synthesised narratively. A total of 2035 articles were screened and four trials were eligible. Three studies had low risk of bias: one reported a positive effect on our primary outcome of interest, excessive SABA prescribing; another reported positive effects on the ratio of inhaled corticosteroid (ICS)-SABA prescribing, and asthma control; a third reported no effect on outcomes of interest. One study at high risk of bias reported a reduction in exacerbations and primary care consultations. There is some evidence that electronic alerts reduce excessive prescribing of SABAs, when delivered as part of a multicomponent intervention in an integrated health care system. However due to the variation in health care systems, intervention design and outcomes measured, further research is required to establish optimal design of alerting and intervening systems.

## Introduction

Asthma affects an estimated 300 million individuals worldwide and almost 30 million people below 45 years of age in Europe.^1^ With a prevalence of 6% in 2016–2017^2^ and an estimated 5.4 million people receiving treatment,^3^ asthma is the most common long-term condition in the United Kingdom (UK).^4^ In 2015-2016 there were approximately 1.4 million hospital admissions for asthma in England and Wales^5^ and whilst the number of asthma deaths has fallen by five percent from 2015 to 2016, this remains higher than the 15-year average.^6^

The National Review of Asthma Deaths (NRAD) identified that, of 195 deaths from asthma between 2012 and 2013, 39% of those who died were prescribed more than 12 short-acting beta_2_-agonist inhalers (SABAs) in the previous year, with 4% prescribed more than 50 SABAs in the same time period.^7^

Frequent use of SABAs is an internationally recognised marker of poor control^8^ and a potentially modifiable warning sign of impending serious asthma attacks^9–12^ and asthma death.^13–17^ Asthma control is defined as the extent to which the manifestations of asthma, commonly wheeze, shortness of breath, chest tightness, cough and variable expiratory airflow limitation, can be observed in the patient, or have been reduced or removed by treatment.^18,19^ Control can be assessed by current symptoms and future risk of adverse outcomes;^8^ patients with good asthma control have less need for SABAs and require no emergency visits.^20^ Following the National Review of Asthma Deaths, the electronic surveillance of prescription refill frequency was recommended to alert clinicians to people with asthma prescribed excessive quantities of SABAs.^7^

General practice computer systems increasingly use reminders and alerts for preventative care and disease management^21,22^ including asthma.^23,24^ Computer decision support systems (CDSSs), defined as ‘active knowledge systems which use two or more items of patient data to generate case-specific advice,’^25^ have the potential to influence prescribing behaviour. Efforts to automate reminder systems and improve efficiency in both prevention and chronic disease management have yielded some improvements when assessed using randomised trials.^26^ However, evaluations suggest CDSSs do not consistently improve prescribing behaviour and clinical outcomes^27^ and the role of electronic alerts to identify and reduce excessive SABA prescribing remains unclear. This review aims to provide a systematic overview of the extent to which electronic alerts in primary care computer systems can identify excessive prescribing of SABAs, and assess the impact of these interventions on SABA prescribing, asthma management and asthma control.

## Results

### Study selection

**Fig. 1:**
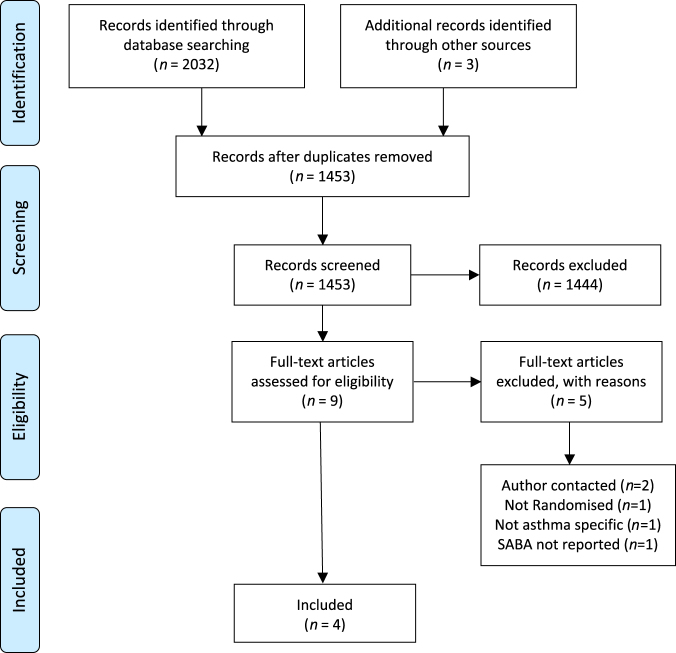
PRISMA flow chart

Fig. 1 details the systematic search and eligibility assessment. From 2035 titles, four studies were selected as eligible.^28–31^ No ongoing or unpublished trials were identified. Risk of bias is reported in Table 1.

**Table 1:** Risk of bias

Study	Selection bias	Allocation concealment bias	Performance bias	Detection bias	Attrition bias	Selective reporting	Other bias	Overall risk
McCowan et al.^28^	No	No	No	Unclear: blinding of outcome assessors not detailed	Yes: attrition rate variation was not fully explained. No intention-to-treat analysis	Unclear: no protocol	No	C-High
Eccles et al.^29^	No	No	No	No	No	No	No	A-Low
Zeiger et al.^30^	No	No	No	No	No	No	No	A-Low
Tamblyn et al.^31^	No	No	No	No	No	No	No	A-Low

### Study characteristics

RCTs were conducted between 2001 and 2015; two recent studies (published in 2014 and 2015) were carried out within integrated healthcare systems in the United States^30^ and Canada^31^ respectively, whilst two older studies were from the United Kingdom (published in 2001 and 2002).^28,29^ The features of interventions are summarised in Table 2. A detailed description of the interventions can be found in Supplementary Appendix 1. Methods of alerting included computerised prompts,^28,29^ an electronic message to physicians^30^ and a dashboard alert.^31^ Three of the four studies included people with asthma under-18 years of age, of which the lower age range for inclusion was 5 years of age,^31^ 12 years of age^30^ and one not reported.^28^ No studies stratified findings by age range. The characteristics of studies are presented in Table 3.

**Table 2:** Summary of intervention features

Features in addition to alert	McCowan et al.^28^	Eccles et al.^29^	Zeiger et al.^30^	Tamblyn et al.^31^
Guideline decision support				
Allergy Specialist Referral				
Specialist asthma nurse home-care monitoring				
Self-management plan				
Patient advice sheet				
Patient information letter

**Table 3:** Characteristics of included studies

Author (Country)	Study design	Participant and setting	Age (years)	Time scale	Inclusion criteria	Exclusion criteria
McCowan et al.^28^ (2001, UK)	Cluster RCT	46 clusters: 46 practice, 447 patients	All; Avg yrs Inv: 32.6 Ctl: 37.4	6 Months, No baseline data	All ages, on asthma register	Not specified
Eccles et al.^29^ (2002, UK)	Cluster RCT with 2 × 2 incomplete block design	62 clusters: 62 primary care practices; 5139 patients	> = 18 years	24 Months; 12 monthsbaseline, 12 months intervention	General practices in north east of England; 50% of doctors using EMIS or AAH Meditel system to view clinical data/issue prescriptions during consultations	Single-handed practices
Zeiger et al.^30^ (2014, USA)	Randomised stratified block design	Managed care organisation; 1999 patients	12-56 years; Avg yrs Inv: 36.2 Ctl: 36.1	20 Months; 8 months intervention; 12 months follow-up	12– 56 years physician diagnosed asthma; (ICD code: 493) in previous 3 years, > = 7 SABAs dispensed, continuous health-plan membership and pharmacy benefit in the prior year, > = 1 ICS canister dispensed in prior 6 months	Excluded co-morbidities coded in the prior year; COPD, emphysema, CF, chronic bronchitis, bronchiectasis, Churg Strauss syndrome, Wegener granulomatosis, Sarcoidosis, pulmonary hypertension, steroid-dependant asthma. Omalizumab in prior 3 months, required an interpreter
Tamblyn et al.^31^ (2015, Canada)	Cluster RCT	Primary care practices. 81 physician clusters; 4447 patients	> 5 years (8.2% aged 5–18 years)	33 Months	> 5 years, asthma diagnosis (ICD9 code: 493), insured through provincial drug plan	COPD diagnosis (ICD9: 491,492, 494,496)

*RCT* randomised control trial, *COPD* chronic obstructive pulmonary disease, *ICD* international classification of diseases, *SABAs* short-acting beta_2_-agonists, *ICS* inhaled corticosteroid, *CF* cystic fibrosis, *INV* intervention, *CTL* control, *Avg yrs* average years of age

### Primary outcome

A brief summary of findings is presented in Table 4. A detailed description of findings can be found in Table 5.

**Table 4:** Summary of findings

Study	Study-defined excessive SABA prescribing	SABA prescribing	ICS prescribing	ICS-SABA prescribing ratio	ICS-LABA prescribing	Asthma reviews	Asthma Exacerbations	Asthma Exacerbation requiring oral steroids	Unscheduled primary care consultations for asthma	Unscheduled secondary care consultations for asthma	Asthma control
McCowan et al.^28^			+/−			+/−	+	+/−	+	+/−	
Eccles et al.^29^		+/−	+/−					+/−	+/−		
Zeiger et al.^30^	+	+	+/−		+			+/−		+/−	
Tamblyn et al.^31^				+							+

*Key:* + positive effect, +/− no effect

*SABA* short-acting beta_2_- agonist, *ICS* inhaled corticosteroid, *LABA* long-acting beta_2_-agonist, *ED* emergency department visit

**Table 5:** Detailed description of findings

Study	Risk of bias	CDSS use	Process outcomes of interest	Clinical outcomes of interest	Interpretation
McCowan et al.^28^	High	Not reported	No between-group difference in number of patients prescribed maintenance therapy based on British asthma guidelines step; *p* *=* 0.51. No between-group difference in the number of patients attending practice initiated asthma reviews OR 0.69 (CI 0.21–2.21).	Fewer exacerbations were reported in the intervention group; 12/147 (8%) in comparison to the control group 57/330 (17%); OR 0.43 (CI 0.21–0.85). Fewer patients were prescribed oral steroids for an exacerbation; 7/147 (5%) of the intervention group compared to 35/330 (11%) of the control group OR 0.42 (CI 0.14–1.29). Fewer primary care consultations were initiated by patients; 22% intervention group compared to 34% control group, OR 0.59 (0.37–0.95). No between-group difference in hospital admissions; OR 0 (CI 0–3.44); or emergency department visits*;* OR 0 (CI 0–9.16).	Of the 46 practices registered to participate, 21 were randomised but only 5 completed the trial due to software problems. Patients treated with CDSS initiated less asthma consultations and were less likely to experience an exacerbation. However it was not clear how exacerbation was defined. The CDSS was not integrated and usage rate was not captured.
Eccles et al. ^29^	Low	Median number of active interactions between groups was zero.	No between-group difference in numbers of SABA prescribed; OR 1.04 (CI 0.83–1.31).No between-group difference in numbers of ICS prescribed; OR 0.95 (CI 0.78–1.16).	No between-group difference in number of consultations for asthma OR 0.94 (CI 0.81–1.08). No between-group difference in number of patients prescribed oral steroids before and after OR 1.0 (CI 0.82–1.22).	This cluster study design with practices as the unit of randomisation, consisted of two arms, asthma and angina each acting as control for the opposite arm e.g., CDSS care for angina acted as control data for asthma CDSS care. Data analysed 12 months before and after. A high number of practices participated (62); prescribing data was obtained from 1139 patients treated with the intervention and 1385 controls. Process of care data was obtained from 1200 patients treated with the intervention and 1163 controls. The intervention had no effect on process or clinical outcomes. Median intervention usage was zero. Data was analysed on an intention to treat basis.
Zeiger et al.^30^	Low	Not reported	^a^Less patients in the intervention group dispensed excessive SABA: 50.7% vs 57.1% control group; RR 0.89, *p* = 0.007 (CI 0.82-0.97) and increased time to be dispensed SABA excessively; HR 0.80; *p* = < 0.001 (CI 0.71–0.91). Greatest effects seen in those with no prior asthma specialist care. Reduction in SABA inhalers dispensed to intervention group at 3 months *p* = 0.002, 6 months *p* = < 0.001, 12 months *p* = < 0.001. Increase in ICS-LABA inhalers dispensed to intervention patients without prior asthma specialist care; 3 months *p* *=* 0.004, 6 months *p* *=* *<* 0.001, 12 months *p* = 0.03.	No between group difference in number of patients with an exacerbation requiring oral steroids; *p* = 0.71, either with or without prior specialist asthma care; *p* *=* 0.38 vs. *p* *=* 0.83. No between group difference in number of patients with an asthma exacerbation requiring > = two oral steroid courses; *p* = 0.55, either with or without prior specialist asthma care; *p* *=* 0.89 vs. *p* *=* 0.50. No between group difference in number of asthma ED visits and/or hospitalisation; *p* = 0.96, either with or without prior specialist asthma care; *p* = 0.55 vs *p* = 0.66.	Real-time outreach intervention in the Kaiser Permanente Southern California (KPSC) managed healthcare system. Usual care included KSPC extensive integrated asthma care management. The intervention reduced excessive SABA use, and ICS/LABA use. Greatest effect were seen in the subgroup of patients without prior asthma specialist care. Physician engagement was not captured as electronic message presented automatically and did not require physician action. Multicomponent intervention included a clinician message, patient letter and allergy referral.
Tamblyn et al.^31^	Low	Physicians did not use the CDSS intervention ‘Asthma Decision Support’ in 60.5% of consultations for patients with out-of-control asthma	Increased ICS-SABA^b^ mean ratio in the intervention group; mean difference = 0.27 *p* = 0.034 (CI 0.02–0.51);	Reduction in out of control asthma events in the intervention group rate difference −8.7/100 PY; *p* = 0.29 (CI −24.7, 7.3). Greatest effects were seen in the sub-group of patients with out-of-control asthma when beginning the study. Rate difference: −28.4, *p* *=* 0.04 (CI −55.6,−1.2); The greatest reduction was seen in the subgroup with out-of-control asthma at beginning of the study when treated with CDS alone had Rate difference: −36.9/100 PY; *p* *=* 0.01.	81 physicians were randomised to ‘asthma decision support;’ 2273 patients treated with the intervention and 2174 controls. This intervention increased the mean ratio ICS-SABA use and reduced the rate of out-of-control asthma episodes. Greatest effect were seen in the subgroup of patients with out-of-control asthma at study entry as treated by CDS alone. In 60% of out-of-control visits the decision support was not accessed by clinicians. No data was available on use over time.

*CI* 95% confidence intervals, *SABA* short acting beta_2_-agonist, *ICS* inhaled corticosteroid, *LABA* long acting beta_2_-agoinst, *RCT* randomised control trial, *I* intervention, *C* Control, *OR* Odds ratio, *RR* risk ratio, *ED* emergency department, *PY* patients per year, *CDSS* computer decision support system

^a^primary outcome of interest

^b^ Reported as fast-acting b-agonist (FABA)

#### Study-defined excessive SABA prescribing

Zeiger et al.^30^ reported a reduction in the number of patients being dispensed excessive SABAs (*p* *=* 0.007) and an increase in length of time between SABA prescriptions (*p* = < 0.001). These effects were noted in the subgroup of patients without prior asthma specialist care who received the intervention (*p* = < 0.001). Tamblyn et al.^31^ reported excessive SABA (expressed as fast-acting b-agonist) dispensing as a composite primary outcome – the rate of out-of-control asthma episodes–which included emergency department (ED) attendances and hospitalisations. It was therefore not possible to determine the effect of the intervention on SABAs alone.

### Secondary outcomes

#### SABA prescribing

Zeiger et al.^30^ reported a reduction in the number of SABAs dispensed at 3 months (*p* = < 0.001), 6 months (*p* = < 0.001) and 12 months (*p* = < 0.001) in the subgroup of patients without prior specialist asthma care. Eccles et al.^29^ reported no significant effect of a computerised decision support system on SABA prescription in the 12 months before and after the intervention (odds ratio (OR) 1.04, 95% CI 0.83–1.31).

#### ICS prescribing

Zeiger et al.^30^ reported no difference in the number of patients dispensed ICS (not as a combination inhaler), whilst Eccles et al.^29^ reported no difference in the number of patients prescribed ICS before and after the intervention. McCowan et al.^28^ reported no between-group difference in maintenance prescribing patterns and no difference in the proportion of patients classified by management step.

#### Ratio of ICS-SABA prescribed

Tamblyn et al.^31^ reported an increase in the ratio of ICS-SABAs dispensed (mean difference (MD) 0.27, *p* = 0.03; 95% CI 0.02–0.51) with higher ratios reported in both subgroups of patients whose asthma was controlled and out of control at the start of the study. Zeiger et al.^30^ reported a controller (ICS) to total medication ratio of greater or equal to 0.5 at 3, 6 and 12 months, in particular for those without prior asthma specialist care. As the ICS to total medication ratio was calculated by the number of ICS canisters or 30-day supplies of oral controller medications dispensed, divided by the total number of controller units and SABA inhalers, it was not possible to determine the ICS-SABA ratio specifically.

#### ICS/LABA prescribing

Zeiger et al.^30^ reported an increase in the number of patients in the subgroup without prior asthma specialist care dispensed an ICS-LABA inhaler at 3 months (*p* = 0.004), 6 months (*p* = < 0.001) and 12 months (*p* = 0.03).

#### Asthma reviews

McCowan et al.^28^ reported no reduction in the number of patients attending practice-initiated asthma reviews.

#### Study-defined asthma exacerbations

McCowan et al.^28^ observed a reduction in asthma exacerbations, with 8% of patients who received the intervention reporting an acute asthma exacerbation compared to 17% in the control group (OR 0.42; 95% CI 0.21-0.85). However, there was no difference in the use of oral steroids to manage these attacks in the intervention and control group. Zeiger et al.^30^ reported no difference in the numbers of patients prescribed oral steroids for an exacerbation irrespective of prior asthma specialist care status. Neither McCowan et al.^28^ nor Zeiger et al.^30^ explicitly defined an asthma exacerbation. Eccles et al.^29^ reported no difference in the numbers of patients prescribed oral steroids before and after the intervention but did not specifically report asthma exacerbations.

#### Unscheduled consultations for asthma

Eccles et al.^29^ found no between-group reduction in the number of primary care asthma consultations, whilst McCowan et al.^28^ reported that patients who received the intervention initiated fewer primary care consultations (OR 0.59; 95% CI 0.37–0.95). However neither study clarified whether consultations were scheduled or unscheduled. Both McCowan et al.^28^ and Zeiger et al.^30^ reported no effect of the intervention on ED attendances or hospitalisations for asthma. Tamblyn et al.^31^ reported ED visits and hospitalisations for asthma as a composite outcome defined as ‘rate of out-of-control asthma episodes,’ therefore secondary care consultations for asthma could not be specifically determined.

#### Asthma control

Tamblyn et al.^31^ reported a reduction in the rate of out-of-control asthma events, defined as a composite outcome of excessive SABA use, ED attendance and hospitalisations for asthma, in the sub-group of patients whose asthma was out-of-control at the beginning of the study (MD −28.4, *p* = 0.04; 95% CI −55.6, −1.2). When stratified by intervention component, the rate of out-of-control asthma events further reduced when patients were treated with CDSS alone (rate difference (RD) −36.9/100 per year, *p* = 0.01) in comparison to those threated with both CDSS and the asthma home care monitoring programme (RD -28.4, *p* = 0.04; 95% CI −55.6,−1.2).

## Discussion

### Main findings

Given the few studies identified, the evidence to support the use of alerts to reduce excessive SABA prescribing in primary care is limited but promising. This review found that electronic alerts, when delivered as a multicomponent intervention in an integrated health care system, have the potential to successfully identify and reduce excessive SABA prescribing. The greatest effect on our outcomes of interest occurred when an alert, delivered in an integrated health care system, flagged excessive SABA prescribing to clinicians and prompted/facilitated intervening actions including referral to an allergy specialist and a patient information letter.^30^ None of the studies included used a SABA alert as a sole intervention.

### Interpretations in relation to published literature

Our findings support previous research on the use of computer decision support for long-term conditions including asthma, chronic obstructive pulmonary disease, diabetes and osteoporosis which found that interventions consisting of multiple components are associated with greater improvement in outcomes than single-target interventions with fewer components.^24,32,33^

There is however no consensus definition on excessive SABA use in the literature. Definitions of excessive SABA use vary from three or more SABAs per quarter^34^ to 12 or more SABAs a year.^7^ Of the two included studies in which excessive SABA use was reported, definitions varied from greater or equal to seven canisters per year (at least four puffs per day per year)^30^ to greater than 250 doses of SABA in the past 3 months.^31^

The identification and reduction of excessive SABA use in Zeiger et al.^30^ study was facilitated by alerts that were not restricted to point-of-care presentation. Such methods of alerting may offer a solution to the dilemma that automatic provision of decision support at point of decision making neither guarantees clinician uptake or engagement^27^ nor predicts improvements in process of care or patient outcomes.^35^ The two studies that showed greatest effects on our outcomes of interest were those carried out recently (in the past three years), in which decision support was integrated with electronic health record (EHRs).^30,31^ Although research has indicated that advice presented within EHRs is less likely to improve care or outcomes than stand-alone programmes,^35^ our findings support the evidence that computer decision support integrated with clinician workflow is associated with improved outcomes.^22,32^ Zeiger et al’s finding of a reduction of excessive SABA use supports the evidence that electronic health records and electronic messaging in an integrated health care system increases clinician adherence to evidence-based guidelines.^36^

In one study, users failed to engage with decision support^29^ whilst in another, clinicians failed to interact with the CDSS in approximately 60% of cases.^31^ However it is not clear whether levels of engagement were consistent between clinicians and whether clinician interaction declined over time. There may be valid reasons to account for the variability in decision support engagement which include technical design of the CDSS, the setting in which the system is deployed and the characteristics of users and the patients treated.^27^ The higher user engagement in Tamblyn et al.^31^ is likely due to the increased ease of use associated with more recent, sophisticated decision support integrated within a comprehensive EHR system that accesses pharmacy, as well as primary and secondary care data. This is in comparison to older interventions, such as that of Eccles et al.^29^ where the intervention was not integrated with the EHR, and in which pharmacy and secondary care data was not captured.

Alerts integrated within EHRs may interrupt clinician workflow and result in “alert fatigue” with up to 96% of alerts over ridden or ignored in one study.^37^ Following user feedback, Eccles et al.^29^ altered decision support to trigger when a clinician entered a relevant morbidity code rather than being automatically activated upon entering a patient’s medical record. Whilst this may have been an attempt to minimise alert fatigue it did not improve CDSS user interaction. It is likely that very low CDSS interactions reflected clinical guidelines being located in a separate system not supported within clinician workflow.

Qualitative research used in conjunction with RCTs has the potential to beneficially influence intervention design and delivery^38^ however none of the included studies reported using qualitative methods to complement intervention design. Such methods may optimise alert design, improve clinician interaction with decision support and aid the interpretation of results.

### Strength and limitations

As interventions to improve prescribing volumes/rates do not necessarily result in more ‘appropriate’ prescribing or improved patient outcomes^27^ both process and clinical outcomes were assessed in this review. However few studies met our inclusion criteria, with only one study reporting our primary outcome of interest. Due to the limited number of published reports of randomised controlled trials in our analyses, there may be possibility of publication bias or selective reporting. Interventions in the two older studies^28,29^ were poorly described which may have limited our interpretation of the findings. We were unable to conduct a meta-analysis due to heterogeneity in intervention design and outcomes evaluated. Due to a lack of reporting no conclusions could be made on health economics.

### Implications for clinical care and future research

There is an increased focus on the digitalisation of the NHS in an attempt to improve safety and quality of care.^39^ Recommendations have called for the national use of electronic alerts to identify excessive prescribing of SABAs in the UK.^7,40^ Due to the few studies identified in this review, the role of alerts to reduce excessive SABA prescribing in the UK’s publically funded national health service (NHS) remains unclear. Integrated care can take many forms involving collaboration between policy providers and commissioners and between service providers, however benefits arise primarily when clinical teams and services are brought together and incentives are aligned to support service improvement.^36^ It is likely that a combination of design, technical capabilities and variety of intervention components, when delivered in an integrated health care system, facilitated the improvements to SABA prescribing and asthma management identified in recent studies. In a publicly funded health care system such as the NHS it remains challenging to deliver such improvements. However this review identifies a number of areas where potential exists and where further research is recommended.

In the UK, 78% of bronchodilators are issued on repeat prescription^41^ yet research fails to address the use of alerts at this point in the prescribing process. Furthermore, two studies from the UK, carried out over a decade ago, did not integrate interventions within EHRs, in contrast to more recent studies from North America. Future research should consider novel ways to deliver SABA alerts as a sole intervention and/or as part of a multicomponent intervention in primary care. Furthermore, the point in the prescribing process at which a SABA alert will have greatest impact should be explored. Interventions should be trialled both in and outside of the consultation to target clinicians and people with asthma.

Standardised methods for the design and reporting of CDSS interventions are recommended to enable a thorough evaluation of process and clinical outcomes. We support previous recommendations that studies use a taxonomy or framework such as Kawamoto et al.^21^ and Berlin et al.^42^ to theoretically underpin the design and reporting of interventions.^25,43^ An explicitly defined outcome set that includes more standardised endpoints, e.g., excessive SABA prescribing and asthma exacerbations, may help the translation of research findings into clinical practice. We recommend that future studies report on both the implementation process and health economics outcomes associated with CDSS-based alerts. Third party external validation of CDSSs is recommended.^35^ Systems evaluation involving academic-commercial collaborations and user testing should be explored to aid the translational research process. End-users should be involved in the design of alerts to optimise interventions and trial design. Future studies should consider mixed methods designs that incorporate qualitative methods before, during and/or after an RCT. Such methods may help determine the barriers and facilitators to alert usage in practice, as well as assisting in the development of alerts that are transferable to the real-world clinical setting.

## Conclusion

There is some evidence that electronic alerts integrated with EHRs and delivered as part of a multicomponent intervention reduce excessive SABA prescribing. Due to variations in health care systems, intervention designs and outcomes measured, further research is required to determine the effects of alerts on excessive SABA prescribing in a publically funded health system. Future research should determine the point at which novel alerts will most effectively reduce excessive SABA prescribing and be accepted by users.

## Materials and methods

The study design was a systematic review, performed following PRISMA-guidelines.^44^ Methods of analysis and inclusion criteria were specified in advance and documented in a protocol^45^ and registered on PROSPERO (International Prospective Register of Systematic Reviews; www.crd.york.ac.uk/PROSPERO/) with identifier CRD 42016035633.

### Selection criteria

Studies were considered for inclusion in this systematic review according to the following criteria.

#### Participants

Studies that delivered care to adults and/or children with asthma, in a primary care setting. Primary care was defined as healthcare delivered in a community setting, most commonly in general practice, by a clinician, nurse or pharmacist. Non-clinical staff for example administrators and/or receptionists were also included.

#### Intervention

CDSSs were included if they incorporated an alert initiated by the excessive prescribing or dispensing of SABAs for asthma. Alerts used in secondary or tertiary care, for other respiratory conditions that were not asthma were excluded.

#### Comparison

The comparator was ‘usual care.’

#### Outcomes

Our primary outcome of interest was excessive SABA prescribing. Excessive prescribing of SABA was assessed on a study-defined basis. Secondary outcomes of interest included additional measures of prescribing and process of care (future SABA and ICS prescribing, ICS/SABA prescribing ratio, ICS/long-acting beta_2_-agonist prescribing (LABA), asthma reviews), and clinical outcomes (asthma exacerbations with/without oral steroids, unscheduled primary and secondary care asthma consultations, asthma control).

#### Study design

Only randomised controlled trials (RCTs), in any language, were included as they are considered the most rigorous way to evaluate intervention effectiveness.^46^

### Search strategy

We searched Medline, Embase, Cinahl, Scopus and Cochrane Library databases from 1990 to 2016 with the search terms listed in Supplementary Appendix 2. We contacted the authors of included studies to clarify intervention design and outcomes measured where necessary. Ongoing and unpublished trials were searched for using the following websites: https://www.isrctn.com/ and https://clinicaltrials.gov/.

Two authors (SM and CG) independently screened titles and abstracts, assessing them against the inclusion criteria. Both authors reviewed the full text of each potentially eligible paper to determine suitability for inclusion. Disagreements were resolved by discussion and, if necessary, arbitration of a third researcher (ADS, AB, MT).

### Data extraction and quality appraisal

Using a piloted data extraction form, SM and ADS independently extracted the following data from included trials: country, setting, funding, study design, healthcare professional and patient population, features of the CDSS intervention, description of the control group, outcome measures, results and risk of bias assessment. SM and ADS compared data extraction, and disagreements were arbitrated by a third researcher (CG) if necessary.

We assessed the risk of bias in each trial using the seven-criteria approach described in section eight of the Cochrane Handbook for Systematic Reviews of Interventions.^47^

### Data analysis

Due to heterogeneity in the CDSS interventions used and in outcomes measured, we undertook a narrative synthesis.

### Data availability

Authors confirm that all relevant data are included in the paper and/or its supplementary information files.

## Electronic supplementary material

Detailed description of intervention characteristics

Search strategies
