# Supplementary material for: The use of electronic alerts in primary care computer systems to identify the excessive prescription of short-acting beta2-agonists for people with asthma: a systematic review
Source: NPJ Prim Care Respir Med. 2018 Apr 16;28:14. doi: 10.1038/s41533-018-0080-z (PMC5902442; doi:10.1038/s41533-018-0080-z)
Supplement: Supplementary file 1 — Detailed description of intervention characteristics [file 41533_2018_80_MOESM1_ESM.pdf]

SUPPLEMENTARY TABLE

Detailed description of intervention features of included studies; adapted from Kawamoto *et al's.*[21] framework for reporting CDSSs and Berlin *et al's.*[42] computer decision support system taxonomy

Features of CDSSs	McCowan *et al.*[28]	Eccles *et al.*[29]	Zeiger *et al.*[30]	Tamblyn *et al.*[31]
**Context**				
**Clinical setting**	Primary Care (NHS)	Primary care (NHS)	Primary Care (Kaiser Permanente Managed Care Organisation)	Primary Care (Insured Health care system)
**Clinical task**	Asthma management	Asthma management	SABA use	Asthma management
**Unit of optimization**	Process and patient outcomes	Process and patient outcomes	Process and patient outcomes	Process and patient outcomes
**Relation to point of care**	In-consultation	In-consultation	Non-specific	In-consultation
**External behavior modification programs**	Not reported	Not reported	Not reported	Not reported
**Potential barriers**	Double data entry required	Method of intervention trigger	None identified	Non-asthma specific consultation
**Knowledge and data source**				
**Clinical knowledge source**	British Asthma Guidelines	Study developed guidelines	National Asthma Education & Prevention Programme/Global Initiative for Asthma	Canadian consensus guidelines
**Data source**	Manually entered	Electronic health record	Research data warehouse	Electronic health record
**Data coding**	Not reported	Not reported	Not reported	Not reported
**Degree of customisation**	Generic	Generic	Personalised	Personalised
**Update mechanism**	Not reported	Not reported	Not reported	Not reported

Decision Support	McCowan *et al.*[28]	Eccles *et al.*[29]	Zeiger *et al.*[30]	Tamblyn *et al.*[31]
**Type of system**	*Asthma Crystal Byte*<br>Computer decision support based on asthma guidelines, clinical scenarios and reminders.	*Computer decision support system (CDSS)*<br>Asthma management and prescribing suggestions based on guidelines, clinical scenarios and health record information	*Real-time outreach*<br>Real-time identification, notification, and facilitated allergy specialist referral for excessive SABA users.	*Asthma Decision Support (ADS)*<br>A dashboard alert, decision support for evidence-based asthma management and asthma home care and monitoring programme.
**Reasoning method**	Not reported	Not reported	Not reported	Not reported
**Alert activation**	Specific presentation complaints or features would trigger a series of prompts based on a previously determined protocol, which would have included a warning for SABA overuse.	Contextualised prompts including management suggestions were triggered when an asthma morbidity code was entered.	An electronic message within the electronic medical record system was sent to a patient's primary care provider, once-only, when a patient with excessive SABA use was identified.	Alert automatically activates upon opening the medical record of a patient with out-of-control asthma. Out-of-control asthma defined as having had an ER visit or a hospitalisation for respiratory-related problems in the past 3 months and/or the excess use (>250 doses dispensed) of fast-acting b-agonist (FABA) in the past 3 months.
**Clinical urgency**	Not reported	Not reported	Not reported	Not reported
**Recommendation explictness**	Explicit	Explicit	Explicit	Explicit
**Logistical complexity**	Simple	Simple	Simple	Simple
**Response requirement**	None	None	None	None

Information delivery	McCowan *et al.*[28]	Eccles *et al.*[29]	Zeiger *et al.*[30]	Tamblyn *et al.*[31]
**Delivery format**	Electronic	Electronic	Electronic	Electronic
**Delivery mode**	Voluntary	Voluntary	Voluntary	Voluntary
**Integration**	No-the programme was delivered on a 3 ½ inch floppy disk to be installed on participants Microsoft Windows compatible computer desktop to be used for asthma consultations.	No-the CDSS was a programme accessible from within the main computerised operating system of the two suppliers. The guideline was a separate pathway within the clinical system.	Yes-the asthma out-reach used the standard Kaiser Permanente Southern California (KPSC) electronic medical record system and electronic registry that allows physicians access to dispensing data and hospital data in a managed care organisation (MCO).	Yes-the ADS can be accessed from a tab in the electronic health record for intervention physicians or from the dashboard alert, when it appears.
**Explanation availability**	The research team constructed non-judgemental feedback and management suggestions based on British Asthma Guidelines but it is not clear if the rationale behind recommendations was presented to physicians.	It was unclear whether clinicians were informed of the guideline evidence when recommendations were presented or whether the recommendations prompted the clinician to access the separate pathway for the guideline.	Physicians- 'Kaiser Permanente and other groups have documented this amount of albuterol is a sign of uncontrolled asthma…'<br>Patients- 'Kaiser Permanente and other groups have shown that care by allergists helps to improve asthma control'; 'too much use of [reliever] may indicate your asthma control could be better.'	Dashboard alert: 'since the last time you accessed the record, new information is available.'<br>Decision support for evidence-based asthma management: provides physicians with access to Canadian asthma guidelines, including translation into assessment tools and recommendations.
**Provision of a recommendation not just an assessment**	Prompts included e.g. 'check inhaler technique,' 'review compliance,' 'consider increasing dose of preventer inhaler.'	The system offered suggestions for management including prescribing.	Included recommendations for treatment e.g. commence preventer medication, physician contact and allergy referral.	Patient specific recommendations generated.

Information delivery	McCowan *et al.*[28]	Eccles *et al.*[29]	Zeiger *et al.*[30]	Tamblyn *et al.*[31]
**Recommendations executed by noting agreement i.e. click 'OK'**	Not reported	Not reported	Not applicable	Dashboard alert- 'Click here to enrol your patient in the Asthma Assist Program immediately'; 'update suggested treatment'; 'proceed with changes.'<br><br>Decision support- patient specific recommendations generated and once selected, prescriptions and action plans where automatically generated.
**Request documentation of reasons if CDSS recommendations not followed**	Not reported	Not reported	Electronic message was sent to physicians informing of the patient's uncontrolled asthma status but did provide recommendations for the physician to act upon.	The dashboard alert could be exited however no further information was reported.
**Interactive delivery**	Yes	Yes	Not applicable	Yes
**Additional clinical data entry not required**	No- the system used data entered on a specialised data entry screen.	Yes-the system anticipated clinicians' requirements by using the information contained within a patient's computerised record.	Yes-patients were identified using computer algorithm data collected in the KPSC research data warehouse. No clinician input was necessary.	Yes-patient's asthma control was determined dynamically, based on a daily retrieval of newly dispensed prescriptions and physician visit information from the provincial health insurance databases at the Regie de l'assurance maladie Quebec (RAMQ).

Workflow	McCowan *et al.*[28]	Eccles *et al.*[29]	Zeiger *et al.*[30]	Tamblyn *et al.*[31]
**System user**	General practitioner	Doctor or nurse	Physician	Physician
**Target decision maker**	General practitioner	Doctor or nurse	Physician and patient	Physician
**Data input intermediary**	General practitioner	Doctor or nurse	Not required	Not required
**Output intermediary**	Not required	Not required	Not required	Not required
**Workflow integration**	Intervention software ran alongside practice software	To access guidelines the clinical system had to be exited	Generated within KPSC electronic medical record system	Integrated with clinician workflow
**Auxiliary features**				
**Local user involvement in development process**	The Asthma Crystal Byte software was designed and developed by a project team, reviewed over an 18 month period by a steering group; 'The General Practitioners in Asthma Group.'	Not reported	Not reported	Not reported
**Provision of decision support to patients as well as providers**	Customised self-management plans and patient advice sheets	Not reported	Physicians were contacted by electronic message and patients by letter	Asthma action plans were automatically generated when recommendation accepted
**CDSS accompanied by periodic performance feedback**	Not reported	Feedback regarding the intervention was obtained from practices at 4 months, however not clinician performance	Not reported	Not reported
**CDSS accompanied by education/training**	'On-line help was incorporated into the software and an installation booklet and user guide were also provided.'	Two members from each intervention practice were sent to a one-day workshop on how to use the system	Not reported	Not reported
**Funding**	Not reported	£900,000 from NHS, EMIS and the Department of Health	Genentech Inc.	Institutes of Health Research and Health Infostructure Partnership Program of Health
