# Supplementary material for: The use of electronic alerts in primary care computer systems to identify the excessive prescription of short-acting beta2-agonists for people with asthma: a systematic review
Source: NPJ Prim Care Respir Med. 2018 Apr 16;28:14. doi: 10.1038/s41533-018-0080-z (PMC5902442; doi:10.1038/s41533-018-0080-z)
Supplement: Supplementary file 2 — Search strategies [file 41533_2018_80_MOESM2_ESM.pdf]

SUPPLEMENTARY METHODS: Search Strategies

Medline

1	exp Asthma/
2	asthma*.mp.
3	(antiasthma* or anti-asthma*).mp.
4	Respiratory Sounds/
5	wheez*.mp.
6	Bronchial Spasm/
7	bronchospas*.mp.
8	(bronc* adj3 spasm*).mp.
9	bronchoconstrict*.mp.
10	exp Bronchoconstriction/
11	(bronch* adj3 constric*).mp.
12	Bronchial Hyperreactivity/
13	Respiratory Hypersensitivity/
14	((bronchial* or respiratory or airway* or lung*) adj3 (hypersensitiv* or hyperreactiv* or allerg* or insufficiency)).mp.
15	((dust or mite*) adj3 (allerg* or hypersensitiv*)).mp.
16	1 or 2 or 3 or 4 or 5 or 6 or 7 or 8 or 9 or 10 or 11 or 12 or 13 or 14 or 15
17	exp DECISION SUPPORT SYSTEMS, CLINICAL/
18	exp DECISION SUPPORT SYSTEMS, MANAGEMENT/
19	exp DECISION SUPPORT TECHNIQUES/
20	exp DECISION MAKING, COMPUTER-ASSISTED/
21	exp MEDICAL RECORDS SYSTEMS, COMPUTERIZED/
22	"decision support".ti,ab
23	"expert system$".ti,ab
24	cdss.ti,ab
25	17 or 18 or 19 or 20 or 21 or 22 or 23 or 24
26	exp Clinical Trial/
27	(randomized or randomised).ab,ti.
28	placebo.ab,ti.
29	dt.fs.
30	randomly.ab,ti.
31	trial.ab,ti.

32 groups.ab,ti.
33 Or/26-32
34 Animals/
35 Humans/
36 34 not (34 and 35)
37 33 not 36
38 16 and 25 and 37
39 Limit 38 to yr=”1990 – 2-16”

Embase

1 exp Asthma/
2 asthma*.mp.
3 (antiasthma* or anti-asthma*).mp.
4 Respiratory Sounds/
5 wheez*.mp.
6 Bronchial Spasm/
7 bronchospas*.mp.
8 (bronc* adj3 spasm*).mp.
9 bronchoconstrict*.mp.
10 exp Bronchoconstriction/
11 (bronch* adj3 constric*).mp.
12 Bronchial Hyperreactivity/
13 Respiratory Hypersensitivity/
14 ((bronchial* or respiratory or airway* or lung*) adj3 (hypersensitiv* or hyperreactiv* or allerg* or insufficiency)).mp.
15 ((dust or mite*) adj3 (allerg* or hypersensitiv*)).mp.
16 1 or 2 or 3 or 4 or 5 or 6 or 7 or 8 or 9 or 10 or 11 or 12 or 13 or 14 or 15
17 exp DECISION SUPPORT SYSTEMS, CLINICAL/
18 exp DECISION SUPPORT SYSTEMS, MANAGEMENT/
19 exp DECISION SUPPORT TECHNIQUES/
20 exp DECISION MAKING, COMPUTER-ASSISTED/
21 exp MEDICAL RECORDS SYSTEMS, COMPUTERIZED/

22	"decision support".ti,ab
23	"expert system$".ti,ab
24	cdss.ti,ab
25	17 or 18 or 19 or 20 or 21 or 22 or 23 or 24
26	Randomized Controlled Trial/
27	Randomization/
28	Controlled Study
29	Clinical Trial/
30	controlled clinical trial/
31	Double Blind Procedure/
32	Single Blind Procedure/
33	Crossover Procedire/
34	or/26-33
35	(clinica$ adj3 trial$).mp.
36	((singl$ or doubl$ or trebl$ or tripl$) adj3 (mask$ or blind$ or method$)).mp.
37	exp Placebo/
38	Placebo$.mp.
39	random$.mp.
40	((control$ or prospective$) adj3 (trial$ or method$ or stud$)).mp.
41	(crossover$ or cross-over$).mp.
42	or/35-41
43	34 or 42
44	Exp ANIMAL/
45	Nonhuman/
46	Human/
47	44 or 45
48	47 not 46
49	43 not 48
50	16 and 25 and 49
51	Limit 50 to yr=”1990 – 2016”

Cochrane Central Trials Register

#1 MeSH descriptor Asthma explode all trees

#2 (asthma*)

#3 (antiasthma* or anti-asthma*)

#4 MeSH descriptor Respiratory Sounds, this term only

#5 (wheez*)

#6 MeSH descriptor Bronchial Spasm, this term only

#7 (bronchospas*)

#8 (bronch* near/3 spasm*)

#9 (bronchoconstrict*)

#10 MeSH descriptor Bronchoconstriction explode all trees

#11 (bronch* near/3 constrict*)

#12 MeSH descriptor Bronchial Hyperreactivity, this term only

#13 MeSH descriptor Respiratory Hypersensitivity, this term only

#14 (bronchial* or respiratory or airway* or lung*) near/3 (hypersensitiv* or hyperreactiv* or allerg* or insufficien*)

#15 (dust or mite*) near/3 (allerg* or hypersensitiv*)

#16 (#1 OR #2 OR #3 OR #4 OR #5 OR #6 OR #7 OR #8 OR #9 OR #10 OR #11 OR #12 OR #13 OR #14 OR #15

#17 MeSH descriptor: [DECISION SUPPORT SYSTEMS, CLINICAL] explode all trees

#18 MeSH descriptor: [DECISION SUPPORT SYSTEMS, MANAGEMENT] explode all trees

#19 MeSH descriptor: [DECISION SUPPORT TECHNIQUES] explode all trees

#20 MeSH descriptor: [Decision Making, Computer-Assisted] explode all trees

#21 MeSH descriptor: [Medical Records Systems, Computerized] explode all trees

#22 "decision support"

#23 "expert system$"

#24 cdss

#25 #17 or #18 or #19 or #20 or #21 or #22 or #23 or #24

#26 Randomized Controlled Trial

#27 randomization

#28 Controlled Study

#29 Clinical Trial

#30 Controlled clinical trial

#31 Double Blind Procedure

#32 Single Blind Procedure

#33 Crossover Procedure

#34 (#26 or #27 or #28 or #29 or #30 or #31 or #32 or #33)

#35 clinica* near/3 trial*

#36 (singl* or doubl* or trebl* or tripl*) near/3 (mask* or blind* or method*)

#37 placebo*

#38 random*

#39 (control* or prospective*) near/3 (trial* or method* or stud*)

#40 (crossover* or cross-over*)

#41 (#35 or #36 or #37 or #38 or #39 or #40)

#42 (#34 or #41)

#43 ANIMAL

#44 Nonhuman

#45 Human

#46 (#43 or #44)

#47 (#46 and not #45)

#48 (#42 and not #47)

#49 (#16 and #25 and #48)

<u>CINAHL</u>

S1 (MH "Asthma)

S2 asthma*

S3 antiasthma* or anti-asthma*

S4 (MH "Respiratory Sounds+")

S5 wheez*

S6	bronchospas*
S7	bronch* N3 spasm*
S8	bronchoconstrict*
S9	bronch* N3 constrict*
S10	(MH "Respiratory Hypersensitivity+")
S11	"house dust mites"
S12	S1 or S2 or S3 or S4 or S5 or S6 or S7 or S8 or S9 or S10 or S11
S13	decision support systems, management
S14	decision support systems, clinical
S15	decision support techniques
S16	decision making, computer assisted
S17	computerized patient record
S18	patient record systems
S19	clinical information systems
S20	decision support
S21	expert systems
S22	cdss
S23	S13 or S14 or S15 or S16 or S17 or S18 or S19 or S20 or S21 or S22
S24	(MH “Clinical Trials”)
S25	random*
S26	placebo*
S27	clinical* N3 (trial* or study or studies)
S28	(single* or double* or triple*) N3 blind*

S29 crossover or cross-over*

S30 S24 or S25 or S26 or S27 or S28 or S29

S31 S12 AND S23 AND S30

SCOPUS

All (asthma) AND

All ("clinical decision support") OR

All ("decision support techniques") OR

All ("computer assisted decision making") OR

All ("computerised medical record system") OR

All ("decision support") OR

All ("expert system") OR

All (cdss) AND

All ("randomized controlled trial") OR

All (randomization) OR

All ("controlled study") OR

All ("RCT") OR

All (trial*) OR

All ("clinical trial") OR

All ("controlled clinical trial") AND

DOCTYPE (ar) AND

PUBYEAR >1989 AND

PUBYEAR < 2017
